# Secondary cancer risk after radiation therapy for breast cancer with different radiotherapy techniques

**DOI:** 10.1038/s41598-020-58134-z

**Published:** 2020-01-27

**Authors:** Quanbin Zhang, Jinbo Liu, Ningjian Ao, Hui Yu, Yingying Peng, Liya Ou, Shuxu Zhang

**Affiliations:** 1 Radiotherapy center, Affiliated Cancer Hospital & Institute of Guangzhou Medical University, Guangzhou, China; 2 State Key Laboratory of Oncology in Southern China, Department of Radiology, Sun Yat-sen University Cancer Center, Guangzhou, China; 3 Department of Biomedical Engineering, Jinan University, Guangzhou, China; 4 Guangzhou Medical University, Guangzhou, China

**Keywords:** Breast cancer, Risk factors

## Abstract

The aim of this study was to estimate the radiation-related secondary cancer risks in organs during the treatment of breast cancer with different radiotherapy techniques, such as three-dimensional conformal radiotherapy (3D-CRT), intensity modulated radiotherapy (IMRT), and volumetric modulated arc therapy (VMAT). The treatment plans for 26 patients with breast cancer who received whole-breast irradiation at a dose of 50 Gy included tangential field 3D-CRT with hard-wedges (W-TF), tangential field IMRT (2F-IMRT), multiple field IMRT (6F-IMRT), and double partial arcs (VMAT). Patients were divided into three groups according to the distance between the contralateral breast (CB) and the body of the sternum. Setup error was simulated by moving the isocenter, and the dose distribution was then recalculated without changing the field fluency distribution. Based on the linear-exponential, the plateau, and the full mechanistic dose-response models, the organ equivalent dose and excess absolute risk were calculated from dose-volume histograms to estimate the secondary cancer risks in organs. Compared with 3D-CRT, IMRT and VMAT showed excellent results regarding tumor conformity and homogeneity; however, the low dose volume to organs was considerably higher in 6F-IMRT and VMAT. Secondary cancer risks for 2F-IMRT were comparable or slightly lower than for W-TF, but considerably lower than for 6F-IMRT or VMAT. After setup error simulation, there was a small increase in secondary cancer risk for 2F-IMRT and an increase of 159% and 318% for 6F-IMRT and VMAT, respectively, compared with W-TF. Although these results were obtained in most patients, they did not necessarily apply to every individual. The secondary cancer risks in the CB decreased significantly in correlation with increased distance for all alternative techniques, although they were higher in VMAT and lower in 2F-IMRT regardless of the distance. After setup error simulation, the increased changes in secondary cancer risks in the CB were comparable between 2F-IMRT, 6F-IMRT, and VMAT, suggesting that the secondary cancer risks in the CB mainly depend on radiotherapy techniques and distance, although the effect of setup error cannot be ignored. In the contralateral lung (CL), the secondary cancer risks were almost independent from distance and depended mainly on radiotherapy techniques; they were rarely affected by setup error. VMAT was associated with a higher secondary cancer risk in the CL. For the ipsilateral lung (IL), the secondary cancer risks were higher than those in other organs because the IL receives high doses to achieve tumor control, and they were relatively lower in VMAT. This warrants special consideration when estimating the secondary cancer risk to the IL. The study results suggested that the optimal radiotherapy method for breast cancer should be determined on an individual basis and according to the balance between secondary cancer risks related to anatomic diversity and setup error, which can prevent blind selection of techniques.

## Introduction

Breast cancer is a common and prevalent cancer in women. Adjuvant radiotherapy after breast cancer surgery is currently the most common treatment to improve local control and overall survival, especially for patients with early-stage disease^1^. Advances in radiotherapy for breast cancer, such as three-dimensional conformal radiotherapy (3D-CRT), intensity modulated radiotherapy (IMRT), and volumetric modulated arc therapy (VMAT), have allowed the delivery of increasingly compliant treatments to target volumes, which are always at the cost of higher low-dose baths^2–5^. Concerns have been raised about a potential increase of radiation-induced secondary cancer risk associated with these new technologies, mainly in the contralateral breast and lungs^6,7^. Two large cohort studies reported that second cancers occurring after radiation therapy for breast cancer are located mostly in organs adjacent to the previously treated fields, such as the organs exposed to the highest radiation dose^8,9^. However, because most of the available data are derived from obsolete treatment techniques that were used 20–30 years ago, epidemiological studies cannot accurately estimate the secondary cancer risk.

Based on biological models, the excess absolute risk (EAR) of a second cancer occurring after exposure to radiation can be estimated from dose-volume histograms (DVH). Studies have compared different treatment techniques to determine the EAR after radiation therapy for breast cancer^10–13^. The EARs in the contralateral breast (CB) and the lungs have been compared between IMRT, VMAT, and tangential field techniques^14^. The thoracic size determines the distance between the breast and the body of the sternum, with affects the dose to the CB and lungs, even for the same treatment protocol. Secondary doses are inversely proportional to the distance from the treatment site. However, detailed and accurate data on radiation-induced secondary cancer risks to healthy organs after breast radiotherapy are lacking, and this information could provide guidance for the choice of radiotherapy techniques for breast cancer. On the other hand, because of the effect of respiratory motion, systematic error, and random error, the setup errors in breast cancer may be up to 5 mm or even larger than 5 mm in three dimensional directions during radiation therapy^15^. This introduces variation in the secondary dose to the CB and lungs in breast cancer treated with different modalities. Therefore, the risk of second cancer can increase or decrease, or show unobvious changes. In this study, we calculated and compared the secondary cancer risks associated with 3D-CRT, IMRT, and VMAT for the treatment of breast cancer after considering the effect of setup error and the distance between the breast and the body of the sternum. The concept of organ equivalent dose (OED) was used for the linear-exponential, plateau, and full mechanistic dose-response models^11,13^ to evaluate the secondary cancer risks to the CB, contralateral lung (CL), and ipsilateral lung (IL) after radiation therapy for breast cancer.

## Methods

### Patients

Computed tomography (CT) data of 26 patients who underwent breast cancer surgery were randomly selected from our database for a retrospective planning study. The median age was 41.6 years (range, 30–50 years). The ratio of right-sided to left-sided breast cancer was 1:1. Approval for retrospective analysis of patient data was obtained from the ethics committee of the Affiliated Cancer Hospital and Institute of Guangzhou Medical University.

**Figure 1:**
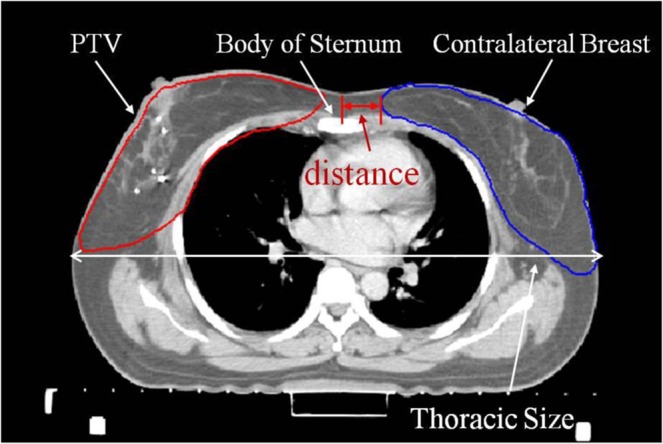
Anatomic distance between the contralateral breast and the body of the sternum.

Patients were divided into three groups according to the distance between the contralateral breast and the body of the sternum (Fig. 1) as follows: patients with a distance of <2 cm were classified into group 1 (G1); patients with a distance of 2–3 cm were classified into group 2 (G2); and patients with a distance of >3 cm were included in group 3 (G3). The G1:G2:G3 patient ratio was 4:5:4. Additionally, each group contained right-sided and left-sided breast cancer.

### Treatment planning

Patients were placed in the supine position with the ipsilateral arm in abduction above the head and scanned with 5 mm slice spacing. The target volumes and organs were delineated by clinical oncologists. The clinical target volume (CTV) included the whole breast. The planning target volume (PTV) was defined as the CTV with a 5–10 mm margin to the delineated target volume to compensate for treatment setup variability and internal organ motion but did not exceed the body. The organs at risk (OARs) included the heart, CB, CL, and IL. The PTV did not include the nodal for irradiation. Tumor bed boosts were not considered at this time.

Four treatment plans were designed for each patient using three different treatment techniques, such as tangential field 3D-CRT with hard-wedges (W-TF), tangential field IMRT (2F-IMRT), multiple field IMRT (6F-IMRT), and double partial arcs (VMAT). W-TF was used for 3D-CRT with hard-wedges (15° or 30°) and consisted of two parallel opposed tangential beams. The gantry and collimator angles were chosen to obtain the best target coverage and completely avoid the CB while minimizing exposure to the IL. The wedge angle, the beam weights, and the leaf openings were manually adjusted to optimize the target coverage and minimize the dose to the OARs. The corresponding 2F-IMRT was generated using the same tangential beams used for 3D-CRT but without wedges. The number of segments for 2F-IMRT was kept as low as possible, generally between 8–15 segments. The minimum segment area was set to 10 cm^2^, and the minimum segment monitor unit was set to 10 monitor units (MU). The 6F-IMRT consisted of multiple co-planar beams including three fields on the outer and inner sides of the breast, such as φ−25°, φ, φ + 25° and ν, ν + 10°, ν + 20°, where the φ and ν on both sides were set according to the tangential field from 3D-CRT. The total number of segments was ≤50, but the segment area and segment monitor unit were the same as those used for 2F-IMRT. The VMAT was created using double coplanar partial arcs with collimator angles of 15° or 345°. One arc was set up in a clockwise (CW) direction from φ to ν, whereas the second arc was performed in a counterclockwise (CCW) direction from ν to φ. The gantry spacing was 4°. The number of iterations for dose calculation did not exceed 100 during the IMRT and VMAT planning processes. All radiotherapy techniques were planned and calculated on the Pinnacle^3^ treatment planning system (TPS, version 9.10, Philips Medical Systems, Fitchburg, WI, USA) using a Collapsed Cone algorithm.

### Evaluation of plans

The total prescribed dose was 50 Gy to the PTV, which was delivered at 2 Gy per fraction^16–18^. The plans were optimized to cover at least 95% of each PTV by administering 95% of the prescribed dose. Dose constraints for the IL were set at V_10_ < 30%, V_20_ < 20%, and V_30_ < 10%, and the average dose D_mean_ < 15 Gy. Dose constrains for the ipsilateral heart were set at V_10_ < 20%, V_20_ < 15%, and V_30_ < 20%, and V_5_ < 15% was set for the contralateral heart with a maximum dose D_max_ < 40 Gy for the spinal cord^16–18^. The V_X_ Gy referred to the volume receiving more than x Gy. Doses to the CB and lungs were kept as low as possible without compromising target coverage. All plans were optimized and evaluated for optimal target coverage, conformity, homogeneity, and dose limits of OARs. The conformity index (CI)^19^, which is a measure of target volume dose distribution conformity, was defined as CI = V_T,ref_/V_T_ × V_T,ref_ /V_ref_, where V_T,ref_ is the target volume covered by reference isodose, V_T_ is the target volume, and V_ref_ is the volume of the reference isodose. A CI closer to 1 indicated better dose conformity. The homogeneity index (HI)^20^, which is a measure of the evenness of dose distribution, was defined as HI = (D_2_–D_98_)/D_50_, where D_2_, D_98_, and D_50_ are the doses covering 2%, 98%, and 50% of the PTV, respectively. HI = 0 is considered the ideal value. Analysis of the OARs included the maximum dose, mean dose, and a set of appropriate define (Vx) and define (Dy) values.

### Secondary cancer risk assessment

Several risk models had been developed to estimate secondary cancer risk, as detailed in Supplementary data. Based on the exported differential DVHs, secondary cancer risks in OARs were calculated using Schneider’s OED concept^13^, which included the impact of fractionation and incorporated a repair and population parameter. The OED was calculated for the linear-exponential, plateau, and full mechanistic dose-response models^10–13^. Assuming that the dose-response was linear to the dose exposition, the linear model OED_*lin*_ for an organ would be calculated as follows:

1 $${{\rm{OED}}}_{lin}=\frac{1}{{{\rm{V}}}_{0}}\sum _{i}{V}_{Di}Di$$

Considering that the probability for cell killing increased exponentially with the dose, which would decrease the risk of cancer induction due to the killing of mutated cells, the linear-exponential model OED_*linear-exp*_ for an organ would be calculated as follows:

2 $${{\rm{OED}}}_{linear-exp}=\frac{1}{{V}_{0}}\sum _{i}{V}_{Di}Di\,exp(-\alpha ^{\prime} Di)$$

If it was assumed that the dose-response had reached a plateau after increasing linearly up to a certain dose because of the balance between cell killing and recovery effects in a fractionated scheme, the plateau model OED_*plateau*_ for an organ would be calculated as follows:

3 $${{\rm{OED}}}_{plateau}=\frac{1}{{V}_{0}}\sum _{i}{V}_{Di}\frac{1-{\exp }(-\alpha ^{\prime} Di)}{\alpha ^{\prime} }$$

When there was an enhancement of the linear exponential model and plateau model and considering the number of fractions in the fractionated therapy, the full mechanistic model OED_*mechanistic*_ for an organ was calculated as follows:

4 $${{\rm{OED}}}_{mechanistic}=\frac{1}{{V}_{0}}\sum _{i}{V}_{Di}\frac{exp(-\alpha ^{\prime} Di)}{\alpha ^{\prime} R}+\left[1-2R+{R}^{2}exp(\alpha ^{\prime} Di)-{(1-R)}^{2}exp\left(-\frac{\alpha ^{\prime} R}{1-R}Di\right)\right]\,$$

The *V*_*Di*_ was the volume of the DVH bin receiving dose *Di*, and V_0_ was the total volume of the organ. For the full mechanistic model, the parameters α′ = 0.044 Gy^–1^ and *R* = 0.15 for a female breast, and α′ = 0.042 Gy^–1^ and *R* = 0.83 for the lungs were derived from a combined fit to the data of atomic bomb survivors and data of Hodgkin’s patients treated with single doses of 2 up to 40 Gy assuming an α/β value of 3 Gy. Similarly, α′ = 0.041 Gy^–1^ for the linear-exponential model and α′ = 0.115 Gy^–1^ for the plateau model were used in regard to a female breast, whereas α′ = 0.022 Gy^–1^ for the linear-exponential model and α′ = 0.056 Gy^–1^ for the plateau model were used in regard to the lungs.

The EAR described the absolute difference in cancer rates between persons exposed to a dose d and those not exposed to a dose beyond the natural dose exposure per 10,000 person-years per Gy. The EAR was calculated as follows^12^:

5 $${\rm{EAR}}={{\rm{EAR}}}_{0}\times {\rm{OED}}$$

EAR_0_ represented the initial slope of the dose-response curve at a low dose and included population-related parameters, such as age at exposure (*agex*), sex (*s*) and attained age (*agea*), namely EAR_0_(*agex*, *s*, *agea*). The EAR of developing a second cancer in one of the investigated organs was calculated as follows:

6 $${\rm{EAR}}={\rm{OED}}\beta ^{\prime} exp[{\gamma }_{e}(agex-30)+{\gamma }_{a}\,\mathrm{ln}(agea/70)]$$

β′ was the initial slope for the dose-response relationship of second cancer induction, γ_*e*_ and γ_*a*_ were the age-modifying factors. All parameters for EAR calculation were from Schneider. All EARs were initially calculated based on patients irradiated at the actual age and assumed to reach an age of 70 years. To remove the EAR variability related to the varying age of irradiated patients, the EAR was recalculated based on all patients who were irradiated at age 30 years and reached an age of 70 years.

### Setup error simulations

The effect of setup error on the variation of secondary doses to organs was simulated by moving the isocenter and recalculating the dose distribution without changing the field fluence distribution^21^. When the isocenter was moved, it was assumed that all beams were incorrectly positioned in the same direction for all fractions during treatment, namely systematic setup error. According to Hurkmans’ work, the systematic setup error in the study was 5 mm in the different directions, such as left–right (L-R), superior–inferior (S-I), and anterior–posterior (A-P). Random errors were ignored at this time.

### Statistical analysis

Statistical analysis was performed using SPSS 19.0 (SPSS Inc., Chicago, IL, USA). Data were expressed as the mean ± standard deviation. Data for dosimetric factors, OEDs, and EARs obtained from different plans were evaluated and compared using Wilcoxon’s signed rank test. Multiple groups of means were compared by one-way analysis of variance (ANOVA) after testing for equality of variance. Homogeneity of variance was assessed using Levene’s test. Differences were considered statistically significant when P < 0.05.

### Ethical approval and informed consent

All experimental protocols used in this study were approved by the Institutional Review Board of Affiliated Cancer Hospital and Institute of Guangzhou Medical University. The requirement for patient informed consent was waived by the Institutional Review Board, and all experiments were performed in accordance with relevant international and national guidelines and regulations.

## Results

### Dosimetry for PTVs and OARs

**Figure 2:**
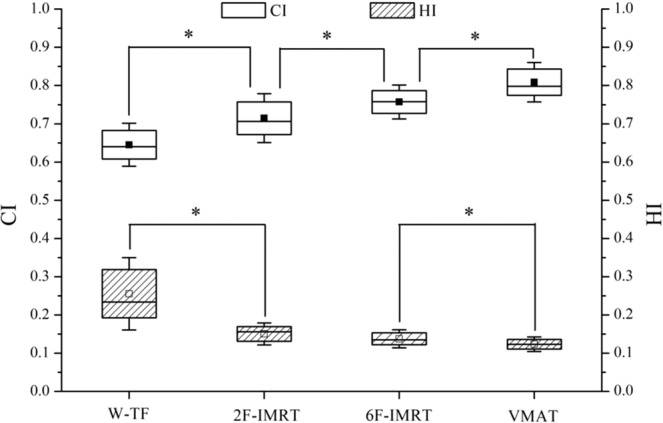
Statistical comparison of conformity index (CI) and homogeneity index (HI) for the PTV in four treatment plans. The midline shows the median; the box covers the 25th to the 75th percentile of the group; the whiskers depict the range of values for standard deviation; a square with or without black fill indicates the mean value. ^*^Indicates a statistically significant difference. W-TF = 3D-CRT with two tangential fields and wedges; 2F-IMRT = IMRT with two tangential fields; 6F-IMRT = IMRT with six fields; and VMAT = VMAT with double partial arcs.

**Figure 3:**
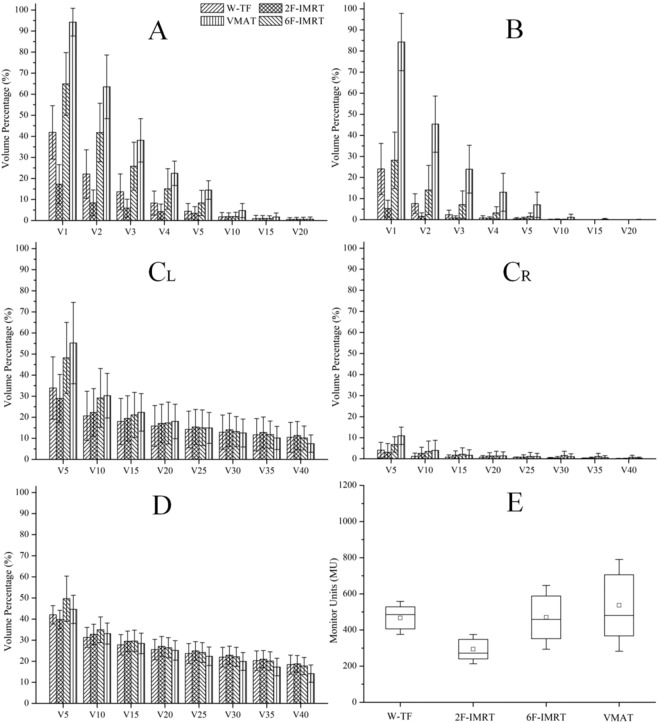
Dosimetric comparison for normal organs in four treatment plans. (**A**) shows the contralateral breast (CB); (**B**) shows the contralateral lung (CL); (**C**_**L**_) shows the ipsilateral heart after irradiation of left-side breast cancer; (**C**_**R**_) shows the contralateral heart after irradiation of right-side breast cancer; (**D**) shows the ipsilateral lung (IL); (**E**) indicates statistical monitor units (MU). V_X_ Gy refers to the volume receiving more than x Gy. W-TF = 3D-CRT with two tangential fields and wedges; 2F-IMRT = IMRT with two tangential fields; 6F-IMRT = IMRT with six fields; and VMAT = VMAT with double partial arcs.

The IMRT and VMAT plans resulted in significantly lower mean doses to OARs and equivalent doses to the PTV than 3D-CRT plans. A representative dose distribution for the four treatment plans is presented in Fig. S1 (Supplementary data). Both 6F-IMRT and VMAT significantly reduced hot spots. The dosimetric parameters and the number of monitor units for all alternative techniques are shown in Figs. 2 and 3. The CI and HI for the PTV were optimal in VMAT, followed by 6F-IMRT, whereas the CI and HI showed the worst values in 3D-CRT’ (P < 0.05). The CI obtained from 6F-IMRT was superior to that from 2F-IMRT, whereas the HI was similar between the two techniques. The minimum low dose volumes to OARs were observed in 2F-IMRT, and VMAT frequently resulted in more low-dose volumes than 2F-IMRT, followed by 3D-CRT and 6F-IMRT (P < 0.05). The low dose volumes from 3D-CRT were similar or inferior to those from 6F-IMRT, whereas the high dose volumes to the IL and heart obtained from VMAT were reduced to some extent. The treatment MUs were lower with 2F-IMRT than with the other three treatment modalities, whereas the treatment MUs were comparable between the other three treatment modalities (P > 0.05).

### Secondary cancer risk

**Figure 4:**
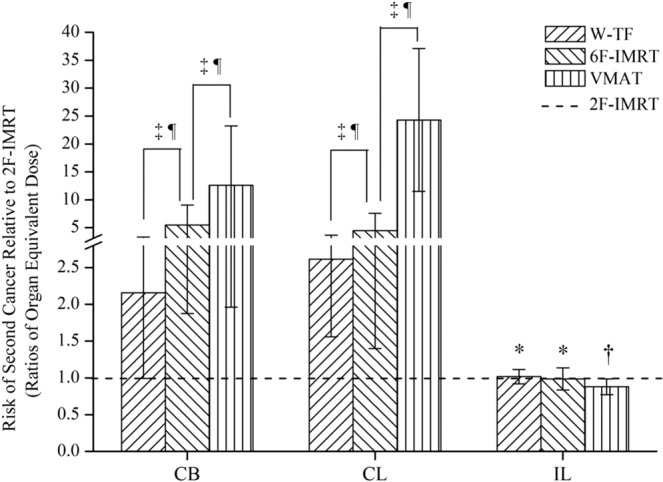
Risk of second cancer relative to 2F-IMRT based on the full mechanistic dose-response model. ^*^Indicates no statistical difference; ^†^indicates significantly lower for alternative techniques compared to 2F-IMRT; ^‡^indicates significantly higher for alternative techniques compared to 2F-IMRT; indicates a statistically significant difference between treatment plans. CB = contralateral breast; CL = contralateral lung; and IL = ipsilateral lung. W-TF = 3D-CRT with two tangential fields and wedges; 2F-IMRT = IMRT with two tangential fields; 6F-IMRT = IMRT with six fields; and VMAT = VMAT with double partial arcs.

**Figure 5:**
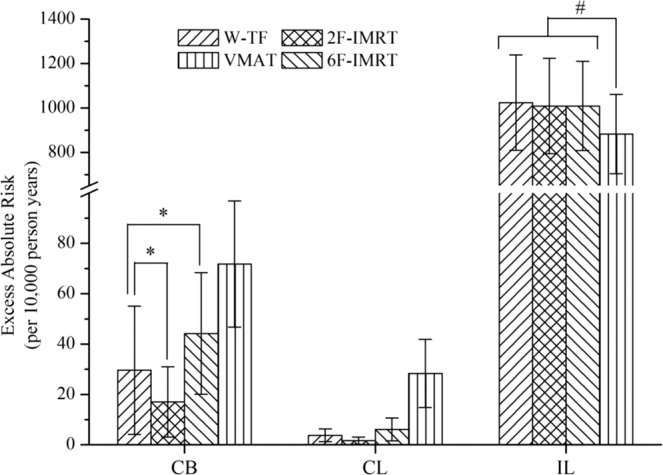
The excess absolute risk (EAR) of radiation-induced secondary cancer based on the full mechanistic dose-response model. ^*^and ^#^indicate a statistically significant difference between treatment plans. CB = contralateral breast; CL = contralateral lung; and IL = ipsilateral lung. W-TF = 3D-CRT with two tangential fields and wedges; 2F-IMRT = IMRT with two tangential fields; 6F-IMRT = IMRT with six fields; and VMAT = VMAT with double partial arcs.

The risks of radiation-induced secondary cancers for all alternative techniques relative to that for 2F-IMRT are shown in Fig. 4. The OEDs to the CB and CL were significantly higher for VMAT than for 2F-IMRT, followed by 6F-IMRT and W-TF. However, the OEDs to the IL were lower for VMAT (P < 0.05) and comparable between the other three treatment modalities (P > 0.05). The corresponding EARs in all organs are shown in Fig. 5. The effect of the age of irradiated patients was eliminated to reduce the variability in EAR. Table S1 shows the OEDs and EARs to organs, which were calculated using linear-exponential and plateau dose-response models (see Supplementary data). The other three alternative techniques had higher secondary cancer risks than 2F-IMRT for most patients. However, when either W-TF or 2F-IMRT was compared with 6F-IMRT or VMAT, the OEDs and EARs for W-TF or 2F-IMRT in all patients were smaller than those of 6F-IMRT or VMAT except in the IL.

**Figure 6:**
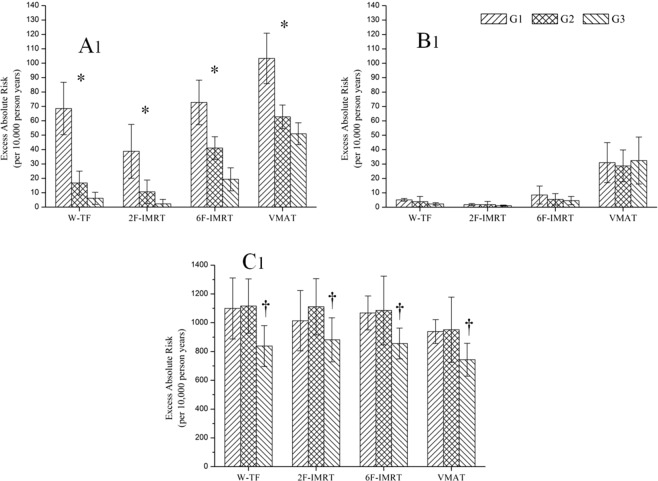
The risk of second cancer in different groups based on the full mechanistic dose-response model. A_1_, B_1_, and C_1_ indicate excess absolute risks to the contralateral breast (CB), contralateral lung (CL), and ipsilateral lung (IL), respectively. ^*^indicates a statistically significant difference between groups; ^†^indicates a significant difference for alternative techniques compared to group 1 (G1) and group 2 (G2). W-TF = 3D-CRT with two tangential fields and wedges; 2F-IMRT = IMRT with two tangential fields; 6F-IMRT = IMRT with six fields; and VMAT = VMAT with double partial arcs.

After adjusting for the effect of the distance between the CB and the body of the sternum, the OEDs and EARs to organs in the different groups are shown in Fig. 6, Table S2, and Table S3. In the CB, the OEDs and EARs decreased significantly in correlation with increased distance for all alternative techniques. The OEDs and EARs to the CB were similar between W-TF and 6F-IMRT when the distance was <2 cm (P > 0.05), whereas the OEDs and EARs were higher for 6F-IMRT than for W-TF when the distance was >2 cm (P < 0.05). For the EARs at different distances, higher secondary cancer risks were observed for VMAT, such as an EAR of 103.311/10,000 PY, whereas secondary cancer risks were lower for 2F-IMRT, such as an EAR of 18.214/10,000 PY. Conversely, secondary cancer risks to the CL were almost independent from distance, and depended mainly on radiotherapy techniques. The EARs to the CL were significantly higher for VMAT than for the other three alternative techniques (P < 0.05), increasing the risks by 6.545-, 16.545-, and 3.664-fold over those for W-TF, 2F-IMRT, and 6F-IMRT, respectively. For the IL, the OEDs and EARs were significantly lower for all alternative techniques when the distance was >3 cm. However, VMAT was associated with lower OEDs and EARs than those of the other three alternative techniques in this case.

### Effects of setup error on secondary cancer risk

The maximum differences of OEDs and EARs to organs for all alternative techniques determined after setup error simulations are shown in Table 1 and Table S4. Compared with W-TF, the OEDs and EARs for the other three treatment modalities showed a greater effect of setup error, especially 2F-IMRT, which changed by 198.82% in the CB and 122.77% in the CL. The OEDs and EARs to the CL showed a lower effect of setup error without significant differences (P > 0.05). There was no significant difference in the increments of change in OEDs and EARs for the CB and IL between 2F-IMRT, 6F-IMRT, and VMAT. In contrast, the increments of change in OEDs and EARs for the CL were significantly different between all alternative techniques, increasing in the order of W-TF, 2F-IMRT, 6F-IMRT, and VMAT.

**Table 1:** The maximum difference of OEDs and EARs in organs after the setup error simulations for all alternative techniques in breast cancer patients, and the data was based on the full mechanistic dose-response model.

Organs	Index	W-TF			2F-IMRT			6F-IMRT			VMAT		
		Max Difference	Δ (%)	P	Max Difference	Δ (%)	P	Max Difference	Δ (%)	P	Max Difference	Δ (%)	P
CB	OED	0.240 ± 0.176	10.03 ± 6.48	>0.05	3.943 ± 3.765	198.82 ± 57.79	<0.05	4.274 ± 3.751	74.21 ± 24.59	<0.05	4.323 ± 2.845	51.16 ± 19.69	<0.05
	EAR	2.209 ± 1.615	N/A	>0.05	36.279 ± 34.636	N/A	<0.05	39.324 ± 34.513	N/A	<0.05	39.770 ± 26.177	N/A	<0.05
CL	OED	0.066 ± 0.034	15.41 ± 6.74	>0.05	0.270 ± 0.249	122.77 ± 66.24	<0.05	0.539 ± 0.347	74.74 ± 31.40	>0.05	1.486 ± 0.981	42.65 ± 22.52	>0.05
	EAR	0.496 ± 0.252	N/A	>0.05	2.026 ± 1.871	N/A	<0.05	4.043 ± 2.606	N/A	>0.05	11.144 ± 7.356	N/A	>0.05
IL	OED	1.828 ± 0.330	0.70 ± 0.58	>0.05	38.376 ± 37.342	29.18 ± 6.64	<0.001	36.222 ± 43.298	29.84 ± 6.81	<0.001	36.064 ± 37.076	34.04 ± 6.93	<0.001
	EAR	7.155 ± 5.779	N/A	>0.05	310.327 ± 89.801	N/A	<0.001	290.219 ± 34.974	N/A	<0.001	290.485 ± 32.831	N/A	<0.001

Note: The difference of OEDs and EARs between pre- and post-simulation in setup error is given by D = D_post_ ‒ D_pre_. The Δ for all alternative techniques results refers to the percentage changes, calculated by Δ = (D_post_ ‒ D_pre)_ / D_pre_ * 100%. P is defined as statistical value between pre- and post-simulation in setup error. OED = organ equivalent dose, and EAR = excess absolute risk. CB = contralateral breast, CL = contralateral lung and IL = ipsilateral lung. W-TF = 3D-CRT with two tangential fields and wedges, 2F-IMRT = IMRT with two tangential fields, 6F-IMRT = IMRT with six fields, and VMAT = VMAT with double partial arcs.

**Figure 7:**
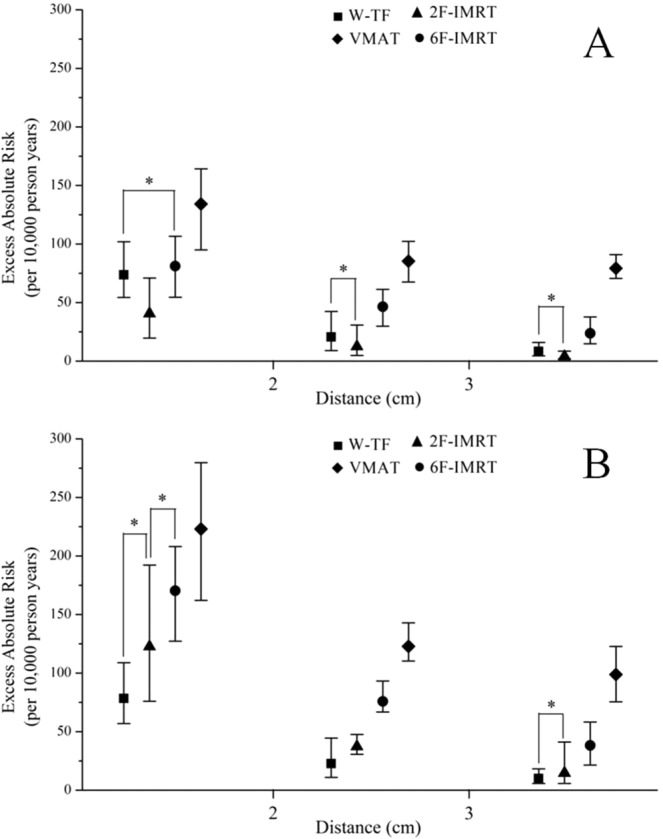
The summation of the excess absolute risks for the contralateral breast (CB) and contralateral lung (CL) obtained at different distances after pre- and post-simulated setup error for all alternative techniques (A for pre-simulation and B for post-simulation). The data were derived from the full mechanistic dose-response model. The geometric symbols show the mean values; the whiskers depict the range of values for minimum and maximum; ^*^indicates no statistically significant difference. W-TF = 3D-CRT with two tangential fields and wedges; 2F-IMRT = IMRT with two tangential fields; 6F-IMRT = IMRT with six fields,; and VMAT = VMAT with double partial arcs.

When considering the distances, the maximum differences of OEDs and EARs to organs after setup error simulations are shown in Table 2, Table 3, Table S5, and Table S6. For the CL, the OEDs and EARs after adjusting for distance showed a lower effect of setup error for all alternative techniques. For the CB and IL, the OEDs and EARs after adjusting for distance also showed a greater effect of setup error except for W-TF. Furthermore, there was a significant difference in the increments of change in OEDs and EARs for the CB at different distances, but not for the IL. If the distance was <2 cm, the change of EAR was 83.168/10,000 PY in the CB after setup error simulation, except for W-TF. If the distance was >3 cm, the effect of setup error on the secondary cancer risk to the CB was significantly decreased, except for VMAT. The total absolute risk of secondary cancer according to distance was determined by adding the EARs to the CB and CL based on pre- and post-simulated setup error for all alternative techniques, as illustrated in Fig. 7. On average, the cumulative EARs for 2F-IMRT were lower before setup error simulation, although there was a small increase in secondary cancer risk for 2F-IMRT and an increase of 159% and 318% for 6F-IMRT and VMAT, respectively, compared with W-TF after setup error simulation. After setup error simulation, if the distance was <2 cm, there was no significant difference in EARs between 2F-IMRT and 6F-IMRT, whereas there was an approximately 38% higher overall EAR for 6F-IMRT than for 2F-IMRT.

**Table 2:** The maximum difference of OEDs in organs obtained from different groups after the setup error simulations for all alternative techniques in breast cancer patients, and the data was based on the full mechanistic dose-response model.

Organs	Index	W-TF		2F-IMRT		6F-IMRT		VMAT	
		Max Difference	P	Max Difference	P	Max Difference	P	Max Difference	P
CB	G1	0.429 ± 0.206	>0.05	8.580 ± 3.224	<0.05	9.040 ± 2.917	<0.001	8.003 ± 1.631	<0.05
	G2	0.169 ± 0.053	>0.05	2.743 ± 0.651	<0.001	2.770 ± 0.890	<0.001	3.132 ± 1.115	<0.05
	G3	0.136 ± 0.065	>0.05	1.072 ± 1.341	>0.05	1.314 ± 0.733	>0.05	2.072 ± 0.872	<0.05
CL	G1	0.092 ± 0.039	>0.05	0.409 ± 0.302	>0.05	0.810 ± 0.351	>0.05	2.024 ± 1.200	>0.05
	G2	0.062 ± 0.027	>0.05	0.280 ± 0.253	>0.05	0.502 ± 0.310	>0.05	1.635 ± 1.361	>0.05
	G3	0.046 ± 0.027	>0.05	0.119 ± 0.099	>0.05	0.312 ± 0.225	>0.05	1.369 ± 1.183	>0.05
IL	G1	1.002 ± 0.244	>0.05	38.864 ± 2.695	<0.05	38.782 ± 3.747	<0.05	38.671 ± 4.016	<0.001
	G2	0.678 ± 0.234	>0.05	38.273 ± 3.585	<0.05	37.797 ± 3.88	<0.05	38.634 ± 2.97	<0.05
	G3	0.637 ± 0.344	>0.05	39.615 ± 8.234	<0.05	39.688 ± 6.800	<0.05	38.908 ± 6.682	<0.05

Note: OED = organ equivalent dose. CB = contralateral breast, CL = contralateral lung and IL = ipsilateral lung. W-TF = 3D-CRT with two tangential fields and wedges, 2F-IMRT = IMRT with two tangential fields, 6F-IMRT = IMRT with six fields, and VMAT = VMAT with double partial arcs. G1 = group 1, G2 = group 2, G3 = group 3.

**Table 3:** The maximum difference of EARs in organs obtained from different groups after the setup error simulations for all alternative techniques in breast cancer patients, and the data was based on the full mechanistic dose-response model.

Organs	Index	W-TF		2F-IMRT		6F-IMRT		VMAT	
		Max Difference	P	Max Difference	P	Max Difference	P	Max Difference	P
CB	G1	3.944 ± 1.893	>0.05	78.935 ± 29.664	<0.05	83.168 ± 26.839	<0.001	73.624 ± 15.009	<0.05
	G2	1.559 ± 0.489	>0.05	22.750 ± 5.990	<0.001	25.485 ± 8.184	<0.001	28.810 ± 10.254	<0.05
	G3	1.253 ± 0.596	>0.05	9.858 ± 12.338	>0.05	12.089 ± 6.744	>0.05	19.067 ± 8.024	<0.05
CL	G1	0.690 ± 0.293	>0.05	3.067 ± 2.264	>0.05	6.075 ± 2.635	>0.05	15.178 ± 9.002	>0.05
	G2	0.461 ± 0.204	>0.05	2.102 ± 1.894	>0.05	3.768 ± 2.328	>0.05	8.514 ± 3.038	>0.05
	G3	0.343 ± 0.149	>0.05	0.893 ± 0.742	>0.05	2.341 ± 1.689	>0.05	10.267 ± 8.870	>0.05
IL	G1	13.513 ± 6.388	>0.05	351.480 ± 149.585	<0.05	290.865 ± 28.100	<0.05	290.035 ± 30.123	<0.05
	G2	5.084 ± 1.758	>0.05	287.046 ± 26.887	<0.05	283.481 ± 29.098	<0.05	289.754 ± 22.273	<0.05
	G3	3.281 ± 2.198	>0.05	297.112 ± 61.753	<0.05	297.658 ± 51.001	<0.05	291.812 ± 50.111	<0.05

Note: EAR = excess absolute risk. CB = contralateral breast, CL = contralateral lung and IL = ipsilateral lung. W-TF = 3D-CRT with two tangential fields and wedges, 2F-IMRT = IMRT with two tangential fields, 6F-IMRT = IMRT with six fields, and VMAT = VMAT with double partial arcs. G1 = group 1, G2 = group 2, G3 = group 3.

## Discussion

In radiation therapy for breast cancer, 3D-CRT with tangential fields, even when used with hard-wedges or dynamic-wedges, is generally used to improve the dose uniformity to the tumor^22^. IMRT and VMAT have been increasingly used to provide a higher conformal dose distribution in the radiation treatment of breast cancer^17^. Compared with 3D-CRT, IMRT and VMAT showed excellent results regarding tumor target conformity and homogeneity, especially 6F-IMRT and VMAT, which is consistent with other studies^17,18^. However, for organs proximal to the fields, such as the CB, CL, IL, and heart, the low dose volumes were considerably higher with 6F-IMRT and VMAT than with 3D-CRT, which are associated with radiation-induced risk of secondary cancer^6^. Along with improved long-term survival rates in breast cancer, the secondary cancer risk after radiation therapy is becoming critical. Approximately 80% of secondary cancers are located either within the radiation field or in the beam-bordering regions^23^. However, accurate prediction of radiation-induced cancer risk is complex because of the lack of long-term follow-up and epidemiological data. Although several risk models have been developed to estimate secondary cancer risks, the incidence rates of radiation-induced cancer are not necessarily a linear function of dose. The dose-risk relationship is linear at lower doses (<2 Gy) for all solid organs and at higher doses (up to 40 Gy) for the breast and lung^24,25^. Previous studies demonstrated that the linear-exponential, plateau, and full mechanistic dose-response models provide a better description of the dose-risk relationship than other linear models for inhomogeneous dose distributions (>2 Gy)^10,12^. However, detailed and accurate data on radiation-induced secondary cancer risks to healthy organs after breast radiotherapy are lacking.

Because the Monte Carlo calculation for 3D-CRT and wedge planning is lacking in commercial planning systems, the Collapsed Cone algorithm was used for all radiotherapy techniques in this study^26^. This may result in inaccuracies in the estimates with respect to the Monte Carlo algorithm^27,28^. In this study, the EARs for 6F-IMRT and VMAT were significantly higher than those for W-TF and 2F-IMRT. These results were consistent with those of previous studies^6,29–31^. VMAT is associated with an especially high risk of secondary cancer relative to multi-field IMRT; similarly, multi-field IMRT is associated with a higher risk of secondary cancer than 3D-CRT without external wedges^30^. By contrast, tangential IMRT has a lower risk of secondary cancer than 3D-CRT with external wedges^31^. This was attributed to the leakage of radiation outside the field, which increased in correlation with increased field numbers and MUs. For example, the MUs were considerably higher for 6F-IMRT and VMAT. The mean secondary cancer risk for 2F-IMRT was slightly lower than that for W-TF, although this was not true for every patient. A similar trend was observed when comparing 6F-IMRT and VMAT. Therefore, when determining which modality has a higher secondary cancer risk, we must consider differences between individual patients. The differences between the linear-exponential, plateau, and full mechanistic dose-response models were smaller, except for the IL. Because the IL constitutes the target volume or is close to the target, it receives high doses to achieve tumor control. The higher doses to the IL lead to larger differences between the models. This warrants special consideration when estimating the secondary cancer risk to the IL. When organs located in the high dose regions are considered in the risk calculations, the leakage radiation dose has a negligible impact on the calculated risks.

For distances of <2 cm, the secondary cancer risks to the CB were similar between W-TF and 6F-IMRT. Because of the Compton scattering of the primary beam in the wedge, 3D-CRT with hard-wedges may result in an additional secondary radiation dose, which may have an equivalent impact on secondary cancer risk to that of leakage radiation from 6F-IMRT. Conversely, for distances >3 cm, the secondary cancer risks to the CB were significant lower for W-TF than for 6F-IMRT because the effect of Compton scattering from the wedge may be much lower than that of leakage radiation from 6F-IMRT. The secondary dose during radiation therapy is primarily derived from leakage and scattering of radiation, which are inversely proportional to the distance from the treatment site^6^. Therefore, the secondary cancer risk to the CB decreases significantly in correlation with increased distance for all alternative techniques, whereas the secondary cancer risk to the CL is almost independent from distance and mainly depends on radiotherapy techniques. 2F-IMRT with tangential fields resulted in the lowest secondary cancer risk to the CB and CL compared with those of the other three techniques. This could be attributed to the tangential technique, which has the optimal configuration with suitable field numbers and MUs and without wedges^32^. However, the importance of individual anatomy cannot be ignored, although the results of this study were true and credible for most patients. Individual organ doses may deviate from the median of the patient cohort, even by more than a factor of two^33^. Regardless of the distance, the use of 6F-IMRT and VMAT instead of 2F-IMRT may increase the secondary cancer risk. In particular, a younger age at exposure is associated with a higher secondary cancer risk. The risk of developing cancer is common among women who are exposed to radiotherapy at an age <40–45 years^34^. The increased risk associated with radiotherapy is limited to younger women exposed to radiation at <45 years of age [relative risk: 1.59, 95% confidence interval (CI): 1.07–2.36], whereas it is not observed in older women (relative risk: 1.01, 95% CI: 0.76–1.35)^35^. The secondary cancer risk to the IL was significantly decreased for all alternative techniques when the distance was >3 cm. Nevertheless, the secondary cancer risk to the IL was greater than the sum of all other site-specific secondary cancer risks. Such a large cancer risk to the IL is not observed in follow-up studies of breast radiotherapy^1^.

Many studies have indicated that the effect of setup error is greater than that of other delivery parameters (such as gantry and collimator angles) in IMRT, especially the effect of respiratory motion^36^. The setup error needs to be considered when estimating the secondary cancer risks to organs during the treatment of breast cancer with different radiotherapy techniques. After the setup error simulations, the effect of setup error on secondary cancer risk to organs was lower for W-TF and higher for IMRT followed by VMAT. However, the increments of change in secondary cancer risk to the CB and IL were comparable between 2F-IMRT, 6F-IMRT, and VMAT. For the CL, the increments of change in secondary cancer risks were higher for VMAT. This suggested that the sensitivity of setup error in the CB and IL, but not in the CL, is similar or equivalent between 2F-IMRT, 6F-IMRT, and VMAT. Considering the anatomic distance, the effect of setup error on secondary cancer risk to the CB decreased with increased distance, whereas the sensitivity of setup error in the CL and IL was almost similar according to distance for all alternative techniques. This suggested that the secondary cancer risk to the CL mainly depends on radiotherapy technique and is rarely affected by setup error, whereas the secondary cancer risk to the CB mainly depends on radiotherapy technique and distance. Before setup error simulation, the cumulative EAR for 2F-IMRT was lower, whereas there was a small increase in cumulative EAR for 2F-IMRT and an increase of 159% and 318% for 6F-IMRT and VMAT, respectively, compared with W-TF after setup error simulation. These results were not consistent with those reported by Ruben *et al*.^37^, who estimated similar secondary cancer risks between 3D-CRT and the 4-field IMRT of 0.25% and 0.26%, respectively, whereas tangential IMRT showed a lower risk of 0.07%. The secondary cancer risk may increase with the use of 6F-IMRT or VMAT techniques, for which younger patients are more vulnerable.

In this study, radiation therapy for breast cancer was planned using a parallel-opposed pair technique. Multi-field IMRT, especially VMAT, is characterized by a high degree of freedom and variable field numbers, which may facilitate adhering to standard dose constraints at the expense of increasing the volume of normal tissues exposed to low-dose radiation. A reduction of peripheral low dose exposure could be achieved by tangential irradiation without external wedges. Variation in secondary cancer risks may also result from differences in optimization regarding the doses to the heart or lung or requirements regarding PTV coverage. Furthermore, much of the variability in secondary cancer risk could be explained by anatomic diversity, especially the distance between the CB and the body of the sternum, because it was closely correlated with the dose to the CB. Lung cancer accounts for approximately 50–70% of solid cancer risk, whereas CB cancer accounts for approximately 2–8% of secondary cancer risk depending on age at exposure (40 or 60 years) and treatment techniques^38^. When the risk of secondary cancer in organs is a major concern during radiation treatment of the whole breast, 2F-IMRT should be the preferred technique when the distance is <2 cm, whereas 6F-IMRT, or even an inferior 3D-CRT, should be the second option for radiation treatment of breast cancer; when the distance is 2–3 cm, 2F-IMRT, or even a superior 6F-IMRT should be the preferred technique; when the distance is >3 cm, 6F-IMRT or even a superior VMAT should be the preferred radiation therapy technique. In particular, when multi-IMRT or VMAT is selected, low dose constraints in the CB and CL should be much stricter during the optimization of the treatment plan.

The present study had several limitations. Firstly, the dose distributions derived in this study were related to a specific geometry of the linac and may not be representative of other linacs. There could be substantial variation in out-of-field doses among different manufacturers, especially for small fields and beam modifiers, such as external physical wedges, internal physical wedges, and dynamic wedges. Secondly, the setup error simulated in this study was within the size of the planning margin. Other situations could yield different results. Further studies are needed to clarify this issue, which is beyond the scope of the present study. Finally, it was difficult to make a straightforward comparison between calculated risks and observed risks obtained from epidemiological studies. Differences in the age of patients at exposure may lead to considerable variation in follow-up periods among patients. Additionally, specific information on radiotherapy techniques is not yet available for large-scale epidemiological studies^9^. Therefore, there may be considerable variation in organ-specific dose distributions from one individual to another when different treatment protocols are used.

## Conclusions

In summary, the present study estimated the secondary cancer risks in organs after whole-breast irradiation using different radiotherapy techniques. The secondary cancer risks for 2F-IMRT were slightly lower or similar to those for W-TF, but considerably lower than those for 6F-IMRT or VMAT. After setup error simulation, there was a small increase in the cumulative EAR for 2F-IMRT, and a significant increase in the cumulative EAR for both 6F-IMRT and VMAT compared with that for W-TF. These results were based on most patients but not necessarily true for every individual. The secondary cancer risk to the CB mainly depended on radiotherapy technique and distance, although the effect of setup error on secondary cancer risk cannot be ignored. For the CL, the secondary cancer risk was almost independent from distance and mainly dependent on radiotherapy technique, and it was rarely affected by setup error. For the IL, the secondary cancer risk was considerably higher than that in other organs because the IL receives high doses to achieve tumor control. This warrants special consideration when estimating the secondary cancer risk to the IL. Selecting the optimal radiotherapy method for breast cancer must be done individually based on the balance between all secondary cancer risks and according to anatomic diversity and setup error, which can prevent blind selection of techniques.

## Supplementary information

Dataset 1.

## Data Availability

Due to ethical and legal restrictions, data for this manuscript are available after formal request to the corresponding author and the ethics committee of Affiliated Cancer Hospital and Institute of Guangzhou Medical Universityt.

## References

[1] Clarke M (2005). Effects of radiotherapy and of differences in the extent of surgery for early breast cancer on local recurrence and 15-year survival: an overview of the randomised trials. Lancet.

[2] Swanson EL (2012). Comparison of three-dimensional (3D) conformal proton radiotherapy (RT), 3D conformal photon RT, and intensity-modulated RT for retroperitoneal and intra-abdominal sarcomas. Int. J. Radiat. Oncol. Biol. Phys..

[3] Haertl PM (2013). Treatment of left sided breast cancer for a patient with funnel chest: volumetric-modulated arc therapy vs. 3D-CRT and intensity-modulated radiotherapy. Med. Dosim..

[4] Moon SH (2009). Dosimetric comparison of four different external beam partial breast irradiation techniques: three-dimensional conformal radiotherapy, intensity-modulated radiotherapy, helical tomotherapy, and proton beam therapy. Radiother. Oncol..

[5] Beckham WA, Popescu CC, Patenaude VV, Wai ES, Olivotto IA (2007). Is multibeam IMRT better than standard treatment for patients with left-sided breast cancer?. Int. J. Radiat. Oncol. Biol. Phys..

[6] Lee B, Lee S, Sung J, Yoon M (2014). Radiotherapy-induced secondary cancer risk for breast cancer: 3D conformal therapy versus IMRT versus VMAT. J. Radiol. Prot..

[7] Hall EJ, Wuu CS (2003). Radiation-induced second cancers: The impact of 3D-CRT and IMRT. Int. J. Radiat. Oncol. Biol. Phys..

[8] Grantzau T, Mellemkjær L, Overgaard J (2013). Second primary cancers after adjuvant radiotherapy in early breast cancer patients: a national population based study under the Danish Breast Cancer Cooperative Group (DBCG). Radiother. Oncol..

[9] De Gonzalez AB (2010). Second solid cancers after radiotherapy for breast cancer in SEER cancer registries. Br. J. Cancer.

[10] Schneider U, Walsh L (2008). Cancer risk estimates from the combined Japanese A-bomb and Hodgkin cohorts for doses relevant to radiotherapy. Radiat. Environ. Biophys..

[11] Schneider U (2009). Mechanistic model of radiation-induced cancer after fractionated radiotherapy using the linear-quadratic formula. Med. Phys..

[12] Schneider U, Sumila M, Robotka J (2011). Site-specific dose-response relationships for cancer induction from the combined Japanese A-bomb and Hodgkin cohorts for doses relevant to radiotherapy. Theor. Biol. Med. Model..

[13] Schneider U, Zwahlen D, Ross D, Kaser-Hotz B (2005). Estimation of radiation-induced cancer from three-dimensional dose distributions: Concept of organ equivalent dose. Int. J. Radiat. Oncol. Biol. Phys..

[14] Abo-Madyan Y (2014). Second cancer risk after 3D-CRT, IMRT and VMAT for breast cancer. Radiother. Oncol..

[15] Batumalai V, Holloway L, Delaney GP (2016). A review of setup error in supine breast radiotherapy using cone-beam computed tomography. Med. Dosim..

[16] Popescu CC (2010). Volumetric modulated arc therapy improves dosimetry and reduces treatment time compared to conventional intensity-modulated radiotherapy for locoregional radiotherapy of left-sided breast cancer and internal mammary nodes. Int. J. Radiat. Oncol. Biol. Phys..

[17] Jin GH (2013). A comparative dosimetric study for treating left-sided breast cancer for small breast size using five different radiotherapy techniques: conventional tangential field, filed-in-filed, tangential-IMRT, multi-beam IMRT and VMAT. Radiat. Oncol..

[18] Zhao H (2015). A comparative dosimetric study of left sided breast cancer after breast-conserving surgery treated with VMAT and IMRT. Radiat. Oncol..

[19] Paddick I (2000). A simple scoring ratio to index the conformity of radiosurgical treatment plans. Technical note. J. Neurosurg..

[20] Kataria T, Sharma K, Subramani V, Karrthick KP, Bisht SS (2012). Homogeneity Index: An objective tool for assessment of conformal radiation treatments. J. Med. Phys..

[21] Samuelsson A, Mercke C, Johansson KA (2003). Systematic set-up errors for IMRT in the head and neck region: effect on dose distribution. Radiother. Oncol..

[22] Baycan D, Karacetin D, Balkanay AY, Barut Y (2012). Field-in-field IMRT versus 3D-CRT of the breast. Cardiac vessels, ipsilateral lung, and contralateral breast absorbed doses in patients with left-sided lumpectomy: a dosimetric comparison. J. Radiol..

[23] Diallo I (2009). Frequency distribution of second solid cancer locations in relation to the irradiated volume among 115 patients treated for childhood cancer. Int. J. Radiat. Oncol. Biol. Phys..

[24] Preston DL (2007). Solid cancer incidence in atomic bomb survivors: 1958–1998. Radiat. Res..

[25] Gilbert ES (2003). Lung cancer after treatment for Hodgkin’s disease: focus on radiation effects. Radiat. Res..

[26] Hasenbalg F, Neuenschwander H, Mini R, Born EJ (2007). Collapsed cone convolution and analytical anisotropic algorithm dose calculations compared to VMC++ Monte Carlo simulations in clinical cases. Phys. Med. Biol..

[27] Howell RM, Scarboro SB, Kry SF, Derek ZY (2010). Accuracy of out-of-field dose calculations by a commercial treatment planning system. Phys. Med. Biol..

[28] Verhaegen F, Seuntjens J (2003). Monte Carlo modelling of external radiotherapy photon beams. Phys. Med. Biol..

[29] Haciislamoglu E (2019). Secondary cancer risk after whole-breast radiation therapy: field-in-field versus intensity modulated radiation therapy versus volumetric modulated arc therapy. Brit. J. Radiol..

[30] Han EY (2016). Estimation of the risk of secondary malignancy arising from whole-breast irradiation: comparison of five radiotherapy modalities, including TomoHDA. Oncotarget.

[31] Joosten A, Matzinger O, Jeanneret-Sozzi W, Bochud F, Moeckli R (2013). Evaluation of organ-specific peripheral doses after 2-dimensional, 3-dimensional and hybrid intensity modulated radiation therapy for breast cancer based on Monte Carlo and convolution/superposition algorithms: implications for secondary cancer risk assessment. Radiother. Oncol..

[32] Smith W (2010). IMRT for the breast: a comparison of tangential planning techniques. Phys. Med. Biol..

[33] Simonetto C (2019). Exposure of remote organs and associated cancer risks from tangential and multi-field breast cancer radiotherapy. Strahlenther. Onkol..

[34] Travis LB (2012). Second malignant neoplasms and cardiovascular disease following radiotherapy. J. Natl. Cancer Inst..

[35] Boice JD, Harvey EB, Blettner M, Stovall M, Flannery JT (1992). Cancer in the contralateral breast after radiotherapy for breast cancer. N. Engl. J. Med..

[36] Xing L (2000). Dosimetric effects of patient displacement and collimator and gantry angle misalignment on intensity modulated radiation therapy. Radiother. Oncol..

[37] Ruben JD (2008). The effect of intensity-modulated radiotherapy on radiation-induced second malignancies. Int. J. Radiat. Oncol. Biol. Phys..

[38] Joosten A, Bochud F, Moeckli R (2014). A critical evaluation of secondary cancer risk models applied to Monte Carlo dose distributions of 2-dimensional, 3-dimensional conformal and hybrid intensity-modulated radiation therapy for breast cancer. Phys. Med. Biol..

