# Supplementary material for: Secondary cancer risk after radiation therapy for breast cancer with different radiotherapy techniques
Source: Sci Rep. 2020 Jan 27;10:1220. doi: 10.1038/s41598-020-58134-z (PMC6985127; doi:10.1038/s41598-020-58134-z)
Supplement: Supplementary file 1 — Dataset 1. [file 41598_2020_58134_MOESM1_ESM.doc]

**Secondary cancer risk after radiation therapy for breast cancer with different radiotherapy techniques**

Quanbin Zhang^1^, Jinbo Liu^2^, Ningjian Ao^3^, Hui Yu^1^, Yingying Peng^1^, Liya Ou^4^, Shuxu Zhang^1^,*

1 Radiotherapy center, Affiliated Cancer Hospital & Institute of Guangzhou Medical University, Guangzhou, China

2 State Key Laboratory of Oncology in Southern China, Department of Radiology, Sun Yat-sen University Cancer Center, Guangzhou, China

3 Department of Biomedical Engineering, Jinan University, Guangzhou, China

4 Guangzhou Medical University, Guangzhou, China

* Corresponding author

**Second Cancer Risk Model**

Several risk models have been developed to estimate secondary cancer risk, such as International Commission on Radiological Protection (ICRP)^1^, Biological Effects of Ionizing Radiation (BEIR) VII committee^2^, United Nations Scientific Committee on the Effects of Atomic Radiation (UNSCEAR)^3^. Considering the stochastic effects, such as cancer and heritable disease, the ICRP model uses tissue weighting factors and nominal risk coefficients to calculate these effects, when the single or annual doses are lower than 100 mSv. The tissue weighting factors reflect the sensitivity of organs to the effects of cancer and heritable disease, and are averaged over sex, age and different populations. For individualized cancer risk assessment, the BEIR VII risk assessment is definitely preferable over the ICRP risk assessment^4, 5^. The ICRP risk assessment provides only a rough approximation of fatal cancer risk, since the tissue weighting factors and nominal risk coefficients relate to radiation detriment and do not directly relate to fatal cancer risk. But BEIR VII risk assessment directly provides model parameters for specific organs of each sex and includes a parameter describing incidence risk with age at exposure and attained age. The BEIR VII model includes the excess absolute risk (EAR) model and the excess relative risk (ERR) model, which depend on the equivalent dose D, the age at exposure (*agex*), the attained age (*agea*) and the sex (*s*). However, the calculation of effective dose is based on the premise of low doses and homogenous dose distributions, neither of which is applicable to radiotherapy^6^. Due to the doses to adjacent organs are high and heterogeneous, simple dose averaging in organs of interest is not a valid method to obtain an effective dose. The concept of organ equivalent dose (OED) to organs of interest is used to calculate a realistic effective dose that would more accurately reflect carcinogenic risk than one relying only on an average dose to each organ. However, the incidence rates of radiation-induced cancer are not necessarily a linear function of dose. The major uncertainty in using OED is the dose-response relationship for carcinogenesis at higher dose^7^. When dose is lower than 2 Gy, the linear no-threshold model applies with good precision. When dose is higher than 2 Gy, there are two extreme possibilities for the shape of the dose-response curve. The curve remains linear^8^, or the curve decreases linearly-exponentially with increasing dose due to cell killing at higher doses^7, 9^. This includes a plateau dose-response model, which lies midway between the other two models, illustrating the repopulation effects from fractionation^9, 10^.

References

1. ICRP Recommendations of the International Commission on Radiological Protection (Users Edition). ICRP Publication 103 (Users Edition) Ann. ICRP 2007; 37: 2-4.
2. BEIR. Health Risks from Exposure to Low Levels of Ionizing Radiation: BEIR VII, Phase 2. National Academy of Science; Washington, DC: 2006.
3. United Nations Scientific Committee on the Effects of Atomic Radiation. Sources and Effects of Ionizing Radiation: UNSCEAR 2006 Report to the General Assembly, with Scientific Annexes. United Nations; New York: 2006.
4. Joosten A, Bochud F, Moeckli R. A critical evaluation of secondary cancer risk models applied to Monte Carlo dose distributions of 2-dimensional, 3-dimensional conformal and hybrid intensity-modulated radiation therapy for breast cancer. Phys Med Biol, 2014, 59(16): 4697.
5. Donovan EM, James H, Bonora M, Yarnold JR., Evans PM. Second cancer incidence risk estimates using BEIR VII models for standard and complex external beam radiotherapy for early breast cancer. Med Phys, 2012, 39(10): 5814-5824.
6. Zwahlen DR, Ruben JD, Jones P, Gagliardi F, Millar JL, Schneider U. Effect of intensity-modulated pelvic radiotherapy on second cancer risk in the postoperative treatment of endometrial and cervical cancer. Int J Radiat Oncol Biol Phys, 2009, 74(2): 539-545.
7. Hall EJ. Intensity-modulated radiation therapy, protons, and the risk of second cancers. Int J Radiat Oncol Biol Phys, 2006, 65(1): 1-7.
8. Sachs RK, Brenner DJ. Solid tumor risks after high doses of ionizing radiation. Proc Natl Acad Sci USA, 2005, 102(37): 13040-13045.
9. Daşu A, Toma-Daşu I, Olofsson J, Karlsson M. The use of risk estimation models for the induction of secondary cancers following radiotherapy. Acta Oncol, 2005, 44(4): 339-347.
10. Davis RH. Production and killing of second cancer precursor cells in radiation therapy: in regard to Hall and Wuu (Int J Radiat Oncol Biol Phys 2003; 56: 83-88). Int J Radiat Oncol Biol Phys, 2004, 59(3): 916.


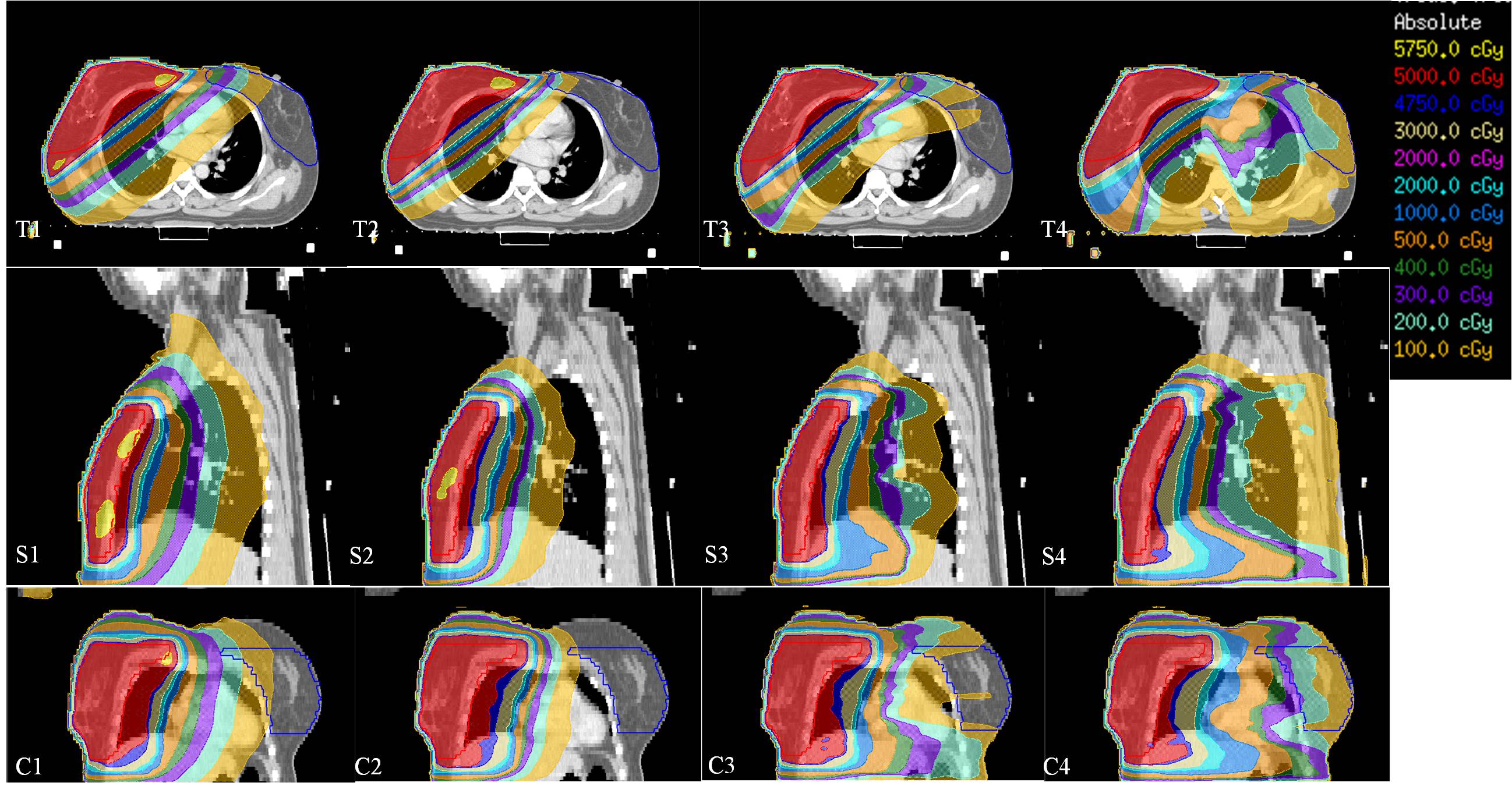


Figure S1 The dose distributions for a representative patient with breast cancer in four types of treatment plans. T stands for transverse, S stands for sagittal and C stands for coronal, while 1, 2, 3 and 4 represent W-TF, 2F-IMRT, 6F-IMRT and VMAT respectively. W-TF = 3D-CRT with two tangential fields and wedges, 2F-IMRT = IMRT with two tangential fields, 6F-IMRT = IMRT with six fields, and VMAT = VMAT with double partial arcs.

Table S1 The risk of second cancer in organs based on the linear-exponential and plateau dose-response model.

| Organs | Index | W-TF | | 2F-IMRT | | 6F-IMRT | | VMAT | |
| --- | --- | --- | --- | --- | --- | --- | --- | --- | --- |
|  |  | linear-exp | plateau | linear-exp | plateau | linear-exp | plateau | linear-exp | plateau |
| CB | OED | 3.295±3.291 | 3.099±3.070 | 1.891±2.101 | 1.758±1.947 | 4.906±2.713 | 4.618±2.514 | 7.961±2.811 | 7.445±2.549 |
|  | EAR | 30.317±30.276 | 28.515±26.245 | 17.399±18.32 | 16.170±15.912 | 45.132±24.956 | 42.486±23.131 | 73.239±25.864 | 68.498±23.450 |
| CL | OED | 0.519±0.350 | 0.512±0.344 | 0.226±0.200 | 0.274±0.308 | 0.849±0.634 | 0.956±0.767 | 3.978±1.942 | 4.343±2.246 |
|  | EAR | 3.992±2.719 | 3.943±2.672 | 1.737±1.556 | 2.104±2.355 | 6.534±4.903 | 7.346±5.783 | 30.592±15.093 | 33.347±17.235 |
| IL | OED | 144.832±30.011 | 138.214±28.733 | 143.298±27.179 | 136.462±26.862 | 143.553±27.179 | 136.704±25.951 | 126.081±25.181 | 119.861±24.006 |
|  | EAR | 1086.238±225.083 | 1036.604±215.496 | 1074.736±210.655 | 1023.464±201.463 | 1076.644±203.843 | 1025.279±194.630 | 945.610±188.858 | 898.959±180.048 |

Note: OED = organ equivalent dose, and EAR = excess absolute risk. CB = contralateral breast, CL = contralateral lung and IL = ipsilateral lung. W-TF = 3D-CRT with two tangential fields and wedges, 2F-IMRT = IMRT with two tangential fields, 6F-IMRT = IMRT with six fields, and VMAT = VMAT with double partial arcs.

Table S2 The OEDs in organs obtained from different groups based on the linear-exponential and plateau dose-response model.

| Organs | Index | W-TF | | 2F-IMRT | | 6F-IMRT | | VMAT | |
| --- | --- | --- | --- | --- | --- | --- | --- | --- | --- |
|  |  | linear-exp | plateau | linear-exp | plateau | linear-exp | plateau | linear-exp | plateau |
| CB | G1 | 7.669±1.903 | 7.175±1.800 | 4.333±2.028 | 4.103±1.895 | 8.159±1.641 | 7.631±1.525 | 11.580±1.709 | 10.729±1.536 |
|  | G2 | 1.839±0.920 | 1.745±0.840 | 1.174±0.898 | 1.096±0.831 | 4.519±0.872 | 4.258±0.805 | 6.909±0.904 | 6.491±0.839 |
|  | G3 | 0.669±0.464 | 0.649±0.445 | 0.310±0.294 | 0.296±0.273 | 2.116±0.878 | 2.037±0.838 | 5.603±0.849 | 5.306±0.747 |
| CL | G1 | 0.709±0.131 | 0.701±0.130 | 0.258±0.111 | 0.254±0.107 | 1.179±0.874 | 1.162±0.859 | 4.354±1.991 | 4.267±1.938 |
|  | G2 | 0.536±0.511 | 0.529±0.501 | 0.260±0.317 | 0.254±0.308 | 0.764±0.553 | 0.751±0.542 | 4.173±1.550 | 4.121±1.519 |
|  | G3 | 0.307±0.116 | 0.304±0.114 | 0.152±0.051 | 0.150±0.050 | 0.620±0.384 | 0.613±0.378 | 4.570±2.366 | 4.471±2.282 |
| IL | G1 | 155.306±29.549 | 148.262±28.323 | 144.074±28.749 | 137.197±27.720 | 152.321±16.664 | 145.114±16.003 | 134.048±11.680 | 127.442±11.106 |
|  | G2 | 157.729±26.458 | 150.554±25.320 | 157.593±27.660 | 150.127±26.308 | 154.183±33.620 | 146.843±32.096 | 135.838±31.857 | 129.161±30.426 |
|  | G3 | 118.881±20.023 | 113.358±19.117 | 125.367±21.594 | 119.329±20.606 | 122.028±15.322 | 116.127±14.484 | 106.407±13.336 | 101.120±15.492 |

Note: OED = organ equivalent dose. CB = contralateral breast, CL = contralateral lung and IL = ipsilateral lung. W-TF = 3D-CRT with two tangential fields and wedges, 2F-IMRT = IMRT with two tangential fields, 6F-IMRT = IMRT with six fields, and VMAT = VMAT with double partial arcs. G1 = group 1, G2 = group 2, G3 = group 3.

Table S3 The EARs in organs obtained from different groups based on the linear-exponential and plateau dose-response model.

| Organs | Index | W-TF | | 2F-IMRT | | 6F-IMRT | | VMAT | |
| --- | --- | --- | --- | --- | --- | --- | --- | --- | --- |
|  |  | linear-exp | plateau | linear-exp | plateau | linear-exp | plateau | linear-exp | plateau |
| CB | G1 | 70.557±17.508 | 66.014±16.557 | 39.865±18.655 | 36.921±17.432 | 75.060±15.100 | 70.204±14.029 | 106.533±15.727 | 98.710±14.131 |
|  | G2 | 16.917±8.463 | 16.053±7.732 | 10.801±8.266 | 10.085±7.642 | 41.578±8.020 | 39.176±7.408 | 63.567±8.313 | 59.721±7.723 |
|  | G3 | 6.156±4.271 | 5.969±4.094 | 2.851±2.704 | 2.720±2.516 | 19.469±8.077 | 18.741±7.711 | 51.551±7.808 | 48.817±6.873 |
| CL | G1 | 5.315±0.9585 | 5.255±0.976 | 1.935±0.830 | 1.901±0.800 | 8.845±6.553 | 8.712±6.442 | 32.652±14.931 | 32.002±14.537 |
|  | G2 | 4.022±3.831 | 3.965±3.757 | 1.948±1.374 | 1.906±1.308 | 5.727±4.145 | 5.633±4.062 | 33.797±11.622 | 33.406±11.389 |
|  | G3 | 2.303±0.867 | 2.280±0.858 | 1.140±0.386 | 1.126±0.376 | 4.652±2.878 | 4.595±2.836 | 34.272±17.743 | 33.536±17.117 |
| IL | G1 | 1164.796±221.614 | 1111.964±212.419 | 1080.559±215.618 | 1028.975±207.903 | 1142.404±124.979 | 1088.355±120.019 | 1005.359±87.596 | 955.817±83.296 |
|  | G2 | 1182.967±198.432 | 1129.154±189.902 | 1181.951±207.451 | 1125.951±197.306 | 1156.374±252.153 | 1101.320±240.719 | 1018.786±238.924 | 968.710±228.195 |
|  | G3 | 891.605±150.170 | 850.185±143.378 | 940.254±161.957 | 894.967±154.544 | 915.209±114.919 | 870.955±108.630 | 798.049±122.519 | 758.400±116.192 |

Note: EAR = excess absolute risk. CB = contralateral breast, CL = contralateral lung and IL = ipsilateral lung. W-TF = 3D-CRT with two tangential fields and wedges, 2F-IMRT = IMRT with two tangential fields, 6F-IMRT = IMRT with six fields, and VMAT = VMAT with double partial arcs. G1 = group 1, G2 = group 2, G3 = group 3.

Table S4 The maximum difference of OEDs and EARs in organs after the setup error simulations for all alternative techniques in breast cancer patients, and the data was based on the linear-exponential and plateau dose-response model.

| Organs | Index | Max difference for W-TF | | Max difference for 2F-IMRT | | Max difference for 6F-IMRT | | Max difference for VMAT | |
| --- | --- | --- | --- | --- | --- | --- | --- | --- | --- |
|  |  | linear-exp | plateau | linear-exp | plateau | linear-exp | plateau | linear-exp | plateau |
| CB | OED | 0.246±0.180 | 0.227±0.166 | 4.058±3.856 | 3.778±3.663 | 4.391±3.833 | 4.082±3.634 | 4.459±2.945 | 4.061±2.706 |
|  | EAR | 2.265±1.655 | 2.086±1.523 | 37.336±35.476 | 34.759±33.702 | 40.398±35.262 | 37.558±33.436 | 41.025±27.096 | 37.361±24.894 |
| CL | OED | 0.069±0.036 | 0.068±0.035 | 0.289±0.272 | 0.280±0.261 | 0.574±0.380 | 0.557±0.364 | 1.603±1.056 | 1.549±1.023 |
|  | EAR | 0.521±0.266 | 0.512±0.261 | 2.166±2.043 | 2.102±1.959 | 4.306±2.848 | 4.176±2.733 | 12.022±7.918 | 11.616±7.670 |
| IL | OED | 1.034±0.901 | 1.000±0.824 | 43.603±12.207 | 41.733±11.807 | 40.596±4.791 | 38.798±4.604 | 40.872±4.585 | 39.018±4.384 |
|  | EAR | 7.753±6.755 | 7.501±6.177 | 327.024±91.551 | 313.001±88.552 | 304.469±35.929 | 290.986±34.528 | 306.538±34.387 | 292.638±32.878 |

Note: OED = organ equivalent dose, and EAR = excess absolute risk. CB = contralateral breast, CL = contralateral lung and IL = ipsilateral lung. W-TF = 3D-CRT with two tangential fields and wedges, 2F-IMRT = IMRT with two tangential fields, 6F-IMRT = IMRT with six fields, and VMAT = VMAT with double partial arcs.

Table S5 The maximum difference of OEDs in organs obtained from different groups after the setup error simulations for all alternative techniques in breast cancer patients, and the data was based on the linear-exponential and plateau dose-response model.

| Organs | Index | Max difference for W-TF | | Max difference for 2F-IMRT | | Max difference for 6F-IMRT | | Max difference for VMAT | |
| --- | --- | --- | --- | --- | --- | --- | --- | --- | --- |
|  |  | linear-exp | plateau | linear-exp | plateau | linear-exp | plateau | linear-exp | plateau |
| CB | G1 | 0.447±0.199 | 0.394±0.210 | 8.863±3.151 | 8.287±3.179 | 9.318±2.798 | 8.715±2.819 | 8.311±1.521 | 7.588±1.489 |
|  | G2 | 0.170±0.054 | 0.167±0.049 | 2.527±0.668 | 2.320±0.618 | 2.833±0.912 | 2.587±0.835 | 3.203±1.147 | 2.903±1.025 |
|  | G3 | 0.137±0.066 | 0.132±0.062 | 1.091±1.372 | 1.019±1.270 | 1.334±0.748 | 1.244±0.686 | 2.116±0.891 | 1.924±0.812 |
| CL | G1 | 0.097±0.041 | 0.095±0.040 | 0.440±0.332 | 0.425±0.317 | 0.854±0.401 | 0.829±0.383 | 2.180±1.290 | 2.107±1.251 |
|  | G2 | 0.064±0.028 | 0.063±0.028 | 0.299±0.275 | 0.290±0.264 | 0.544±0.344 | 0.525±0.327 | 1.230±0.457 | 1.187±0.430 |
|  | G3 | 0.048±0.021 | 0.047±0.021 | 0.126±0.107 | 0.123±0.104 | 0.331±0.243 | 0.323±0.235 | 1.473±1.269 | 1.425±1.233 |
| IL | G1 | 2.047±0.972 | 1.918±0.884 | 49.537±20.132 | 47.472±19.468 | 40.360±3.626 | 38.557±3.461 | 40.803±4.527 | 38.955±4.053 |
|  | G2 | 0.690±0.268 | 0.709±0.259 | 40.260±3.689 | 38.511±3.568 | 39.783±4.055 | 38.038±3.897 | 40.736±3.032 | 38.906±2.935 |
|  | G3 | 0.432±0.307 | 0.431±0.306 | 41.681±8.430 | 39.862±8.167 | 41.807±7.033 | 39.952±6.776 | 41.103±7.009 | 39.217±6.694 |

Note: OED = organ equivalent dose. CB = contralateral breast, CL = contralateral lung and IL = ipsilateral lung. W-TF = 3D-CRT with two tangential fields and wedges, 2F-IMRT = IMRT with two tangential fields, 6F-IMRT = IMRT with six fields, and VMAT = VMAT with double partial arcs. G1 = group 1, G2 = group 2, G3 = group 3.

Table S6 The maximum difference of EARs in organs obtained from different groups after the setup error simulations for all alternative techniques in breast cancer patients, and the data was based on the linear-exponential and plateau dose-response model.

| Organs | Index | Max difference for W-TF | | Max difference for 2F-IMRT | | Max difference for 6F-IMRT | | Max difference for VMAT | |
| --- | --- | --- | --- | --- | --- | --- | --- | --- | --- |
|  |  | linear-exp | plateau | linear-exp | plateau | linear-exp | plateau | linear-exp | plateau |
| CB | G1 | 4.111±1.827 | 3.625±1.936 | 81.543±28.993 | 76.245±29.247 | 85.724±25.738 | 80.173±25.937 | 76.460±13.989 | 69.805±13.703 |
|  | G2 | 1.562±0.500 | 1.532±0.451 | 23.246±6.149 | 21.340±5.686 | 26.062±8.389 | 23.803±7.683 | 29.464±10.554 | 26.709±9.429 |
|  | G3 | 1.263±0.604 | 1.212±0.568 | 10.036±12.624 | 9.374±11.686 | 12.273±6.880 | 11.448±6.311 | 19.464±8.195 | 17.699±7.472 |
| CL | G1 | 0.729±0.310 | 0.715±0.303 | 3.302±2.486 | 3.190±2.375 | 6.405±3.007 | 6.215±2.876 | 16.353±9.673 | 15.802±9.382 |
|  | G2 | 0.481±0.212 | 0.475±0.210 | 2.240±2.064 | 2.177±1.982 | 4.078±2.578 | 3.936±2.452 | 9.224±3.424 | 8.899±3.222 |
|  | G3 | 0.360±0.157 | 0.354±0.154 | 0.943±0.806 | 0.923±0.777 | 2.481±1.824 | 2.424±1.763 | 11.047±9.514 | 10.690±9.250 |
| IL | G1 | 15.355±7.289 | 14.387±6.627 | 371.528±150.990 | 356.039±146.014 | 302.700±27.194 | 289.180±25.955 | 306.025±31.924 | 292.159±30.396 |
|  | G2 | 5.177±2.010 | 5.321±1.946 | 301.949±27.667 | 288.833±26.762 | 298.374±30.412 | 285.283±29.225 | 305.521±22.742 | 291.798±22.009 |
|  | G3 | 3.241±2.304 | 3.233±2.296 | 312.610±63.224 | 298.965±61.255 | 313.551±52.750 | 299.636±50.822 | 308.270±52.564 | 294.127±50.202 |

Note: EAR = excess absolute risk. CB = contralateral breast, CL = contralateral lung and IL = ipsilateral lung. W-TF = 3D-CRT with two tangential fields and wedges, 2F-IMRT = IMRT with two tangential fields, 6F-IMRT = IMRT with six fields, and VMAT = VMAT with double partial arcs. G1 = group 1, G2 = group 2, G3 = group 3.
